# Oral swabs with a rapid molecular diagnostic test for pulmonary tuberculosis in adults and children: a systematic review

**DOI:** 10.1016/S2214-109X(23)00469-2

**Published:** 2023-12-12

**Authors:** E Chandler Church, Karen R Steingart, Gerard A Cangelosi, Morten Ruhwald, Mikashmi Kohli, Adrienne E Shapiro

**Affiliations:** a HIV Vaccine Trials Network, Fred Hutchinson Cancer Center, Seattle, WA, USA; b Division of Allergy and Infectious Diseases, University of Washington, Seattle, WA, USA; c Honorary Research Fellow, Department of Clinical Sciences, Liverpool School of Tropical Medicine, Liverpool, UK; d Department of Environmental and Occupational Health Sciences, School of Public Health, University of Washington, Seattle, WA, USA; e FIND, Geneva, Switzerland; f Department of Global Health, University of Washington, Seattle, WA, USA

## Abstract

**Background:**

Tuberculosis is a leading cause of infectious disease mortality worldwide, but diagnosis of pulmonary tuberculosis remains challenging. Oral swabs are a promising non-sputum alternative sample type for the diagnosis of pulmonary tuberculosis. We aimed to assess the diagnostic accuracy of oral swabs to detect pulmonary tuberculosis in adults and children and suggest research implications.

**Methods:**

In this systematic review, we searched published and preprint studies from Jan 1, 2000, to July 5, 2022, from eight databases (MEDLINE, Embase, Scopus, Science Citation Index, medRxiv, bioRxiv, Global Index Medicus, and Google Scholar). We included diagnostic accuracy studies including cross-sectional, cohort, and case-control studies in adults and children from which we could extract or derive sensitivity and specificity of oral swabs as a sample type for the diagnosis of pulmonary tuberculosis against a sputum microbiological (nucleic acid amplification test [NAAT] on sputum or culture) or composite reference standard.

**Findings:**

Of 550 reports identified by the search, we included 16 eligible reports (including 20 studies and 3083 participants) that reported diagnostic accuracy estimates on oral swabs for pulmonary tuberculosis. Sensitivity on oral swabs ranged from 36% (95% CI 26–48) to 91% (80–98) in adults and 5% (1–14) to 42% (23–63) in children. Across all studies, specificity ranged from 66% (95% CI 52–78) to 100% (97–100), with most studies reporting specificity of more than 90%. Meta-analysis was not performed because of sampling and testing heterogeneity.

**Interpretation:**

Sensitivity varies in both adults and children when diverse methods are used. Variability in sampling location, swab type, and type of NAAT used in accuracy studies limits comparison. Although data are suggestive that high accuracy is achievable using oral swabs with molecular testing, more research is needed to define optimal methods for using oral swabs as a specimen for tuberculosis detection. The current data suggest that tongue swabs and swab types that collect increased biomass might have increased sensitivity. We would recommend that future studies use these established methods to continue to refine sample processing to maximise sensitivity.

**Funding:**

Bill and Melinda Gates foundation (INV-045721) and FIND (Netherlands Enterprise Agency on behalf of the Minister for Foreign Trade and Development Cooperation [NL-GRNT05] and KfW Development Bank, German Federal Ministry of Education and Research [KFW-TBBU01/02]).

## Introduction

Tuberculosis remains a leading cause of infectious disease mortality and is among the top 15 causes of death worldwide.^1^ An estimated 7·5 million people were diagnosed with tuberculosis in 2022, an increase from 2021. There were an estimated 1·3 million deaths from tuberculosis in 2022, with around 167 000 of those deaths occurring in people living with HIV.^1^ In 2022, only 63% of people treated for tuberculosis had a microbiological test confirming the diagnosis.^1^ WHO recommends using a molecular rapid diagnostic test as the initial test for diagnosing tuberculosis in adults and children with signs and symptoms of tuberculosis.^2^

Specimens for diagnosing pulmonary tuberculosis include sputum, gastric aspirates, nasopharyngeal aspirates, urine, and stool. For diagnosing pulmonary tuberculosis using sputum, Xpert MTB/RIF and XPERT MTB/RIF Ultra represent a high sensitivity molecular testing method for adults and children when the specimen can be obtained.^3^, ^4^ Sputum production can be limited in individuals with a weak cough, people living with HIV, and children younger than 5 years. These limitations often require the use of other respiratory specimens (eg, gastric aspirates) in young children or induced sputum in adults, which require medical equipment and specialised staff training.^5^

To improve access to tuberculosis testing, oral swabs have been proposed as a new clinical specimen for pulmonary tuberculosis diagnosis. Oral swab specimens are analysed by nucleic acid amplification tests (NAAT) that amplify *Mycobacterium tuberculosis* DNA collected by the swab. Multiple types of swabs and NAAT platforms are being studied, with PCR the most commonly used NAAT method.^5^ To date, there are no oral swab-based recommendations for tuberculosis detection by WHO.

**Research in context**

**Evidence before this study**

Despite recent advances in tuberculosis diagnostics, most methods still require sputum for testing. However, many populations, including children and people living with HIV, have difficulty producing sputum samples. Obtaining sputum for diagnosis in people who cannot produce it themselves can be resource intensive and require trained staff, which can make obtaining a microbiological diagnosis much more challenging. We conducted a search on July 5, 2022, in the following databases: Medline, Embase, Scopus, Science citation index, medRxiv, bioRxiv, Global index medicus, and Google Scholar using search terms including: ((“Mycobacterium tuberculosis” OR “tuberculosis” OR “TB” OR “pulmonary tuberculosis”) AND (“oral swab*” OR “tongue swab*” OR “buccal swab*”)), for systematic reviews looking at oral swabs. The search was repeated on Aug 8, 2023. We did not find any previous systematic reviews looking at oral swabs for tuberculosis at the time of the original search, and no reviews focusing specifically on oral swab methods are currently in existence, although Savage and colleagues addressed oral swab methods in a review of non-sputum-based diagnostics.

**Added value of this study**

To our knowledge, this is the first comprehensive review of oral swabs as a specimen type for the diagnosis of pulmonary tuberculosis. The methods of the included studies were highly variable, particularly regarding the area of the oral cavity swabbed, the type of swab used, the processing of the swab sample before analysis, and the type of molecular testing performed on the swab. Several studies directly compared one of these factors. This review brings together all the available evidence on oral swabs to help future studies use the techniques that yield the best results in terms of sensitivity and specificity.

**Implications of all the available evidence**

Our study has substantial implications for future research into oral swabs. By bringing together the available evidence, researchers can choose the techniques that will maximise the potential sensitivity and specificity of oral swabs within the study population. We would recommend that researchers focus their efforts on tongue swabs and swab types that collect increased biomass, as these methods appear to increase sensitivity. In addition, although in-house PCR testing was primarily used in the early stages of research on oral swabs, recent studies show that reasonable sensitivity can be achieved using Xpert MTB/Rif Ultra, which is widely used for sputum testing and available to most sites that would benefit from this type of sample.

**Figure 1:**
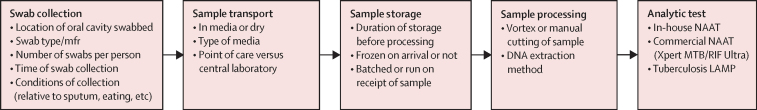
Potential sources of variability in sensitivity and specificity for oral swabs LAMP=loop-mediated isothermal amplification. Mfr=manufacturer. NAAT=nucleic acid amplification test.

Diagnosing pulmonary tuberculosis using oral swabs as a clinical specimen comprises a multi-stage process of sampling, processing, and analysis. Differences in each component of the analytic pathway can contribute to overall variability in diagnostic accuracy of oral swabs (figure 1). There are several potential advantages to this clinical specimen over sputum-based sampling, including possible self-collection and use in populations that can have difficulty producing sputum.^6^ Oral swab samples could also be combined with other sample types such as Determine TB LAM Ag (Abbott, Lake Forest, IL, USA) or gastric aspirate in populations with low bacillary burden.^7^ One potential clinical pathway for the use of oral swabs is shown in the appendix (p 8).

We performed a systematic review of existing oral swab literature to determine the diagnostic accuracy of this emerging sample type, identify sources of variability in accuracy, and characterise priorities for further optimisation and research.

## Methods

### Search strategy and selection criteria

We searched eight databases (MEDLINE, Embase, Scopus, Science Citation Index, medRxiv, bioRxiv, Global Index Medicus, and Google Scholar) for published and preprint studies from Jan 1, 2000, to July 5, 2022, without language restriction (see appendix p 1 for the full search strategy). The review was performed on July 5, 2022. Search strategy included terms for tuberculosis, oral swabs, and brand names for various oral swabs. We reviewed reference lists of included articles and any relevant review articles identified. We also contacted researchers at FIND and other experts in the field of tuberculosis diagnostics (including GAC and MK) for information on unpublished studies. Ethical approval was not required for this review.

We included cross-sectional, cohort, and case-control studies evaluating the sensitivity and specificity of oral swab sampling and molecular testing against a microbiological or composite reference standard for the diagnosis of pulmonary tuberculosis. The microbiological reference standard included solid or liquid culture or a molecular WHO-recommended rapid diagnostic performed on sputum. The composite reference standard combined the microbiological reference standard with clinical criteria including symptoms and chest imaging. We considered both published articles and preprints from which we could extract or derive true positive, false positive, false negative, and true negative values. We only included studies where both the reference standard and test strategy were assessed in all participants. We excluded studies that evaluated saliva as a testing sample as this review is focused on swab-based samples, which are not used for saliva collection. Oral swabs are collected using a porous or scraping material that is rubbed over a specific location in the oral cavity, such as the tongue or buccal mucosa, so that friction lifts surface cellular material to the swab as well as absorbing oral fluids, whereas saliva samples are oral fluids deposited directly into a collection vessel, without use of an intermediary device. Participants were adults (aged 15 years or older) and children (younger than 15 years) who were evaluated for presumptive pulmonary tuberculosis.^8^, ^9^

### Data analysis

We used Covidence systematic review software to manage the selection of studies.^10^ Two review authors independently scrutinised titles and abstracts identified from literature searching to identify potentially eligible studies. We retrieved the article of any citation, identified by any review author, for full-text review. Then, two review authors (ECC and AES) independently assessed articles for inclusion using the predefined selection criteria. We resolved any discrepancies by discussion or with a third review author. We followed review methods described in the Cochrane Handbook for Systematic Reviews for Diagnostic Test Accuracy.^11^

Three review authors (ECC, KRS, and AES) independently extracted data on key participant characteristics and information on the index text (location of oral swabbing, type of swab, and type of NAAT), reference standard, and diagnostic 2 × 2 table data (number of true positives, false positives, false negatives, and true negatives). We used the QUADAS-2 tool, tailored to this review, to assess the quality of the included studies (appendix p 4).^12^ Three review authors (ECC, KRS, and AES) independently assessed all domains for risk of bias and the first three domains for concerns regarding applicability. Study authors were contacted for clarifications and missing data. Review authors who were also authors of included studies did not perform quality assessment or extract data from their own study or studies.

We determined sensitivity and specificity estimates and 95% CIs for individual studies, and generated forest plots using Review Manager 5 (Review Manager 2020). We visually inspected forest plots for heterogeneity.

### Role of the funding source

The funding source had no role in the review of the literature, data abstraction, or quality assessment.

## Results

**Figure 2:**
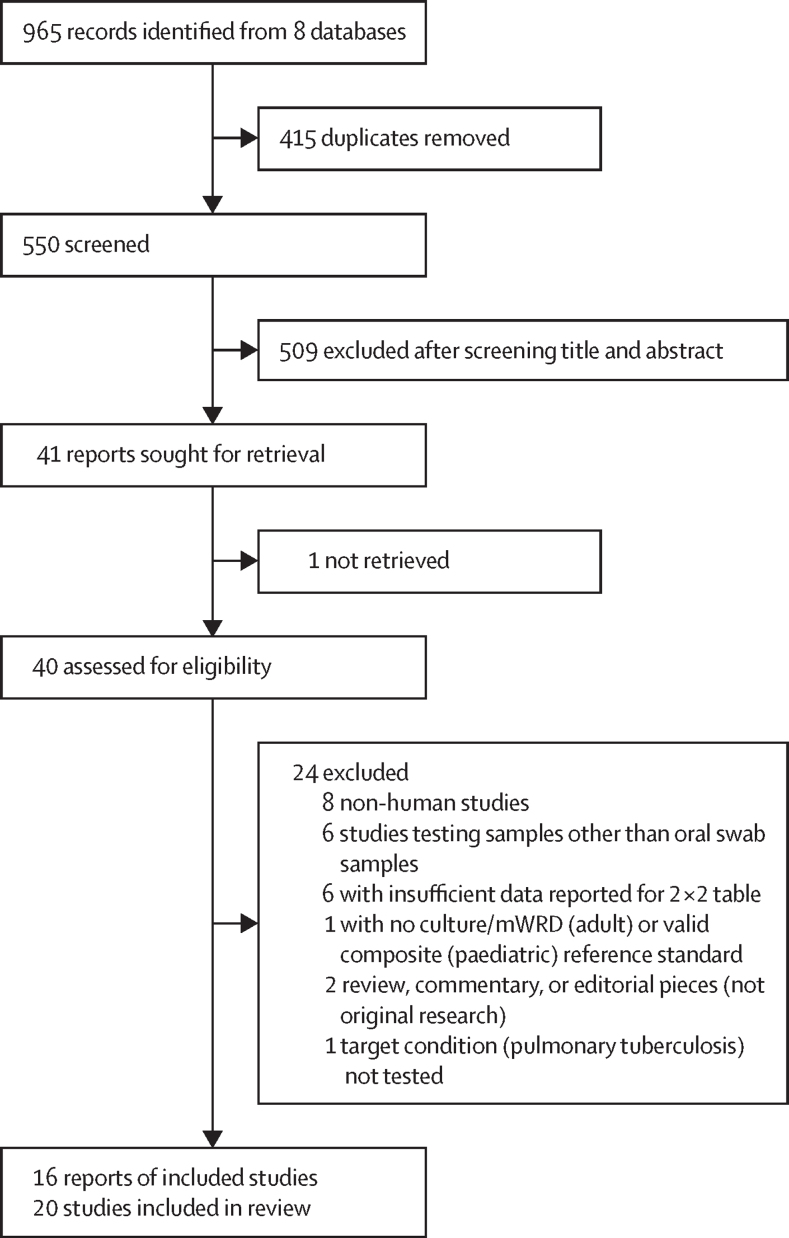
Study selection mWRD=molecular WHO-recommended rapid diagnostic.

We obtained 965 records and, after deleting 415 duplicates, we had 550 records to screen. We excluded 509 records based on title and abstract review and identified 41 for full-text review. We were unable to obtain a full-text copy of one study and were unable to contact the author, so this study was excluded.^13^ We excluded 24 reports due to ineligibility. We identified 16 reports that met our inclusion criteria (figure 2). Four reports reported more than one oral swab testing strategy study, hence we included 20 studies (3083 participants) in the review. Participant characteristics for each study are summarised in the table. When different methods were evaluated in the same study (ie, different swab types), we used the designation a, b; for example, Flores 2020a and Flores 2020b used different swab types and were considered as two distinct studies.

**Table:** Characteristics of oral swabs for the diagnosis of pulmonary tuberculosis by study

	**Population**	**Total participants**	**Median age (years)**	**Children aged <5 years**	**Participants living with HIV (%)**	**Reference standard (primary)**	**Positive by oral swab and reference standard**	**Swab type**	**Number, location of swabs**	**Sample transport**	**Sample storage**	**Laboratory processing**	**Type of NAAT**
Andama 2022, Uganda^22^	Adults aged >18 years presenting for health care with at least 2 weeks of cough	184 (Andama 2022a);37 (Andama 2022b)	33 (IQR 26–43)	..	58 (31·7%)	Sputum Xpert MTB/RIF Ultra	42 (Andama 2022a); 27 (Andama 2022b)	Copan FLOQswab	2–3 tongue swabs	2 swabs placed together in 800 mL of Tris-EDTA buffer. If additional swab collected, stored in a separate container with the same media type	2 swabs processed within 1 h, remaining swab frozen and stored at −80°C until testing	Method a: 2 swabs processed together with Cepheid sample reagent, then prepared for Xpert testing by the same protocol as sputum; method b: 1 swab boiled, incubated, and mixed without use of sample reagent	Xpert MTB/Rif Ultra
Cox 2022, South Africa^30^	Children aged ≤15 years with cough and one other finding suggestive of pulmonary tuberculosis	291	2·7 (IQR 1·2– 6·1)	Not reported	57 (19·6%)	Sputum Xpert MTB/RIF Ultra or culture	20	Puritan Purflock and Copan FloQswab	1–2 buccal or tongue swabs	Sterile saline	Frozen and stored at −80°C until testing	Thawed and 1·6 mL Xpert sample reagent added, vortexed, then incubated	Xpert MTB/Rif Ultra
Ealand 2021, South Africa^21^	Children aged ≤5 years clinically diagnosed with tuberculosis or lower respiratory tract infection	35	0·8 (IQR 0·4– 1·6)	35 (100%)	6 (17·1%)	Clinical diagnosis	11	Copan 480 C swab	1 tongue swab	3 mL Middlebrook 7H9 with OADC and Tween 80	Stored at 4°C	Vortexed with 2 mm glass beads	In-house PCR
Flores 2020, Peru^27^	Children aged <15 years with at least one symptom of pulmonary tuberculosis	288	Not reported	124 (43%)	0	Sputum or gastric aspirate culture	5	Whatman OmniSwab or Whatman EasiCollect FTA card	1 buccal swab	OmniSwab in sterile lysis buffer, EasiCollect dry	OmniSwab frozen and stored at −80°C, EasiCollect at −20°C	OmniSwabs vortexed before freezing, DNA extracted with QIAmp DNA minikit, EasiCollect cut into pieces and placed in basic solution followed by neutral solution to release DNA	In-house PCR (Roche 480 Lightcycler)
Kang 2022, South Korea^20^	Adults aged >18 years with presumptive tuberculosis	272	Mean 58·8 (SD 15·2)	..	1 (0·4%)	Sputum culture	65	OmniGene Oral OMR-110 kit swab	1 gingival swab	Not specified	Frozen and stored at −80°C until testing	Samples liquified and injected into SLIM platform	In-house PCR (Qiagen TAQ PCR Core Kit)
LaCourse 2022, Kenya^23^	Adults aged ≥13 years with presumptive tuberculosis	100	38 (IQR 30–44)	..	54 (54%)	Sputum Xpert MTB/RIF or culture	13	Whatman OmniSwab	2 buccal swabs	Sterile lysis buffer	Frozen and stored at −80°C until testing	Not specified	In-house PCR (IS *6110*-targeted qPCR [Applied Biosystems StepOnePlus Real-Time PCR system])
Lima 2020, Brazil^14^	Screening study in prison inmates, no age restrictions noted	256	Not reported	..	Not reported	Sputum Xpert MTB/RIF	66	Puritan Purflock Ultra swabs	2 tongue swabs 24 h apart	Eppendorf and sterile lysis buffer	1 swab analysed within 24 h, second stored at −80°C for up to 12 weeks	Not specified	Xpert MTB/RIF Ultra
Luabeya 2019, South Africa^19^	Adults aged ≥18 years. Some patients with presumptive tuberculosis, some with confirmed positive Gene Xpert sputum testing	343	35·2 (IQR 25·6–45·8)	..	86 (25·1%)	Sputum Xpert MTB/RIF	74 (Luabeya 2020a); 74 (Luabeya 2020b)	Whatman OmniSwab and Puritan Purflock Ultra	1 tongue swab with Purflock Ultra, 2 tongue swabs with Whatman OmniSwab	Sterile lysis buffer	Frozen and stored at −80°C within 8 h of collection	DNA extracted with QIAmp DNA minikit	In-house PCR (IS *6110*-targeted qPCR [Applied Biosystems StepOnePlus Real-Time PCR system])
Mesman 2019, Peru^15^	Adults diagnosed with pulmonary tuberculosis	63	Not reported	NA	Not reported	Sputum culture	15	Whatman OmniSwab or Whatman EasiCollect FTA card	1 buccal swab	OmniSwab in sterile lysis buffer, EasiCollect dry	Unknown	Not specified	Xpert MTB/RIF Ultra
Mesman 2020, Peru^17^	Adults aged ≥18 years with culture confirmed tuberculosis	123	32 (IQR 23–44)	NA	4 (3·3%)	Sputum culture	38 (Mesman 2020a); 15 (Mesman 2020b)	Whatman OmniSwab or Whatman EasiCollect FTA card	3 buccal swabs	OmniSwab ejected into media, EasiCollect stored dry	OmniSwabs vortexed and stored at −80°C. FTA cards frozen at −20°C	FTA cards cut into pieces and placed in basic solution, then placed in neutral solution. QIAmp DNA minikit used to extract DNA. OmniSwabs warmed and extracted using QIAmp DNA minikit	In-house PCR (Roche 480 Lightcycler)
Molina-Moya 2020, Moldova^31^	Adults with a clinical diagnosis of pulmonary tuberculosis	300	Mean 48·8 (SD 14·4)	NA	0	Sputum Xpert MTB/RIF or culture	29	PrimeSwab flocked swab	1 buccal swab	PrimeStore molecular transport media	Vortexed then kept at room temp for up to 30 days or frozen and stored at −20°C	DNA extracted with PrimeXtract nucleic acid extraction and purification kit	In-house PCR (Roche 480 Lightcycler)
Nicol 2019, South Africa^24^	Children aged <15 years with presumptive pulmonary tuberculosis	165	2·6 (IQR 1·1–6·8)	113 (68·5%)	18 (11%)	Sputum Xpert MTB/RIF or culture or clinical diagnosis	17	GE Healthcare OmniSwab or Puritan Purflock Ultra swab	2 buccal swabs	Sterile lysis buffer	Frozen and stored at −80°C	DNA extracted using QIAmp DNA minikit	In-house PCR
Shapiro 2022, South Africa^7^	Adults aged ≥16 years with HIV and a known pulmonary tuberculosis diagnosis pre-treatment or symptoms of tuberculosis	131	36 (IQR not reported)	NA	120 (92%)	Sputum Xpert MTB/RIF Ultra or culture	42	Copan FLOQswab	2 tongue swabs	0·5 mL sterile Tris-EDTA buffer	Frozen and stored at −80°C until testing	Manually processed and concentrated with ethanol	In-house PCR (IS *6110*-targeted qPCR [Applied Biosystems StepOnePlus Real-Time PCR system])
Song 2021, China^32^	Adults aged >16 years with presumptive pulmonary tuberculosis	101	48·5 (range 17–88)	NA	0	Sputum Xpert MTB/RIF or culture	38	Not specified	3 tongue swabs (morning, spot, and evening)	Sterile normal saline	Not specified	Sample centrifuged then resuspended. Pellet heated at 90°C in extraction solution	TB-LAMP assay
Wood 2015, South Africa^16^	Adults aged ≥21 years with positive sputum Gene Xpert	40	Median 38–45*	NA	0	Sputum Xpert MTB/RIF	18	Whatman OmniSwab	3 buccal swabs	In house lysis buffer	Frozen and stored at −80°C	DNA extracted using QIAmp DNA minikit	In-house PCR (Applied Biosystems StepOnePlus Real-Time PCR system)
Wood 2021, Uganda^18^	Adults aged >18 years with presumptive pulmonary tuberculosis	194	Median 30–34*	NA	36 (25%)	Sputum Xpert MTB/RIF Ultra or culture	44	Puritan Purflock Ultra and Copan FLOQSwabs	2 tongue swabs	Sterile lysis buffer	Frozen and stored at −80°C within 8 h of collection	DNA extracted using QIAmp DNA minikit	In-house PCR (Applied Biosystems StepOnePlus Real-Time PCR system)

NA=not applicable. NAAT=nucleic acid amplification test.

* Depending on subgroup. TB-LAMP=tuberculosis loop-mediated isothermal amplification. Temp=temperature.

Of the 20 studies included, 10 (50%) had low risk of bias in all four QUADAS-2 domains. Seven studies (35%) were considered high risk of bias for patient selection due to using a case-control design.^14^, ^15^, ^16^, ^17^, ^18^, ^19^ One study was considered high risk of bias in the index test domain due to incomplete description of oral swab collection and processing methods, and not stating a prespecified threshold for a positive index test.^20^ One study was considered high concern for applicability for patient selection, as it was carried out in a tertiary care referral hospital; however the study was carried out in a population of children with a relatively high prevalence of HIV (17·1%), both of which are target population characteristics for non-sputum-based diagnostic samples.^21^ All studies were assessed as unclear concern for applicability of the index test as there is no standard methodology defined for using oral swabs for tuberculosis detection. All studies were considered low risk of bias in the reference test domain, and 18 were assessed as low risk of bias in the flow and timing domain.^7,14–24,27,30–32^ Two studies were assessed as unclear risk of bias as the interval between index and reference testing was not specified (see appendix pp 7–10 for further details).^27^

The 20 included studies from 16 reports were conducted in eight countries, including South Africa (seven studies), Peru (five), Uganda (three), South Korea (one), Kenya (one), Brazil (one), China (one), and Moldova (one). In adults, two (10%) studies were conducted in populations with a HIV prevalence of more than 50%.^7^, ^23^ One study used oral swabs as a screening specimen in a high-risk, asymptomatic population and all other studies evaluated oral swabs as a diagnostic specimen for participants with signs or symptoms of pulmonary tuberculosis.^14^ Key elements of the included studies such as type of swab used, number of swabs collected per participant, site of the oral cavity swabbed, sample processing details, and type of NAAT used, are summarised in the table.

**Figure 3:**
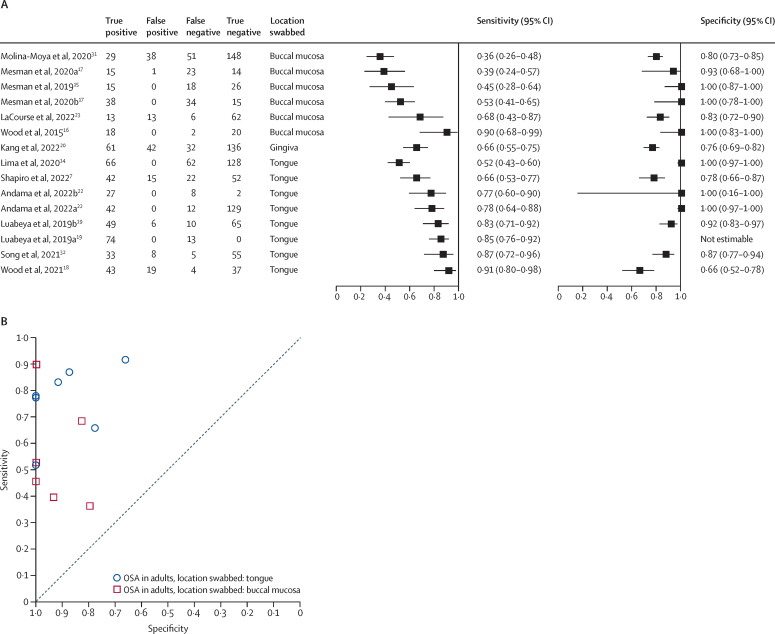
Forest plot (A) and SROC plot (B) of oral swabs in adults using a microbiological reference standard by location of oral cavity swabbed OSA=oral swab analysis. SROC=summary receiver operating characteristic.

15 studies (12 reports, 2223 participants) evaluated oral swabs for tuberculosis detection in adults. Sensitivity of oral swabs and molecular test for the diagnosis of pulmonary tuberculosis in adults against a microbiological reference standard ranged from 36% to 91%, with specificity of oral swabs ranging from 66% to 100% (figure 3A, appendix p 11).

Sensitivity of tongue swabs in adults (eight studies) ranged from 52% to 91%; sensitivity of buccal swabs in adults (six studies) ranged from 36% to 90% (figure 3). The study with the lowest sensitivity for tongue swabs (52%) was performed in asymptomatic individuals as a screening test.^14^ When used as a diagnostic test for individuals with symptoms of pulmonary tuberculosis, sensitivity of tongue swabs in adults ranged from 66% to 92% with specificity ranging from 66% to 100%.

Swab types evaluated included Whatman OmniSwab (six studies [30%]), Copan FLOQSwabs (five studies [25%]), Whatman EasiCollect FTA (two studies [10%]), Puritan PurFlock Ultra (two studies [10%]), multiple swab types (two studies [10%]), PrimeSwab Flocked Swab (one study [5%]), OmniGene Oral OMR-110 Kit (one study [5%]), and unspecified type (one study [5%]). Studies using Copan FLOQSwabs had sensitivities ranging from 66% to 91% and specificity from 66% to 100%; sensitivity using Whatman OmniSwabs ranged from 39% to 90% and specificity from 83% to 100% (appendix p 11).

NAATs used included manual PCR (14 studies [70%]), Xpert MTB/RIF Ultra (five [25%]), and tuberculosis loop-mediated isothermal amplification (TB-LAMP; one [5%]). In-house PCR had sensitivities ranging from 36% to 91% and specificities from 66% to 100%, whereas studies using Xpert MTB/RIF Ultra had sensitivities ranging from 45% to 78% with all specificities reported as 100% (appendix p 11). In the most recent study using Xpert MTB/RIF Ultra, Andama and colleagues achieved a sensitivity of 78% (95% CI 64–88) and a specificity of 100% (95% CI 97–100) using tongue swabbing and new sample processing methods using Cepheid sample reagent.^22^

Four studies in adults reported invalid swab results (13 [1·8%] of 727 total swabs), including six in LaCourse and colleagues, four in Mesman and colleagues, two in Luabeya and colleagues, and one in Andama and colleagues.^15^, ^19^, ^22^, ^23^ Other studies either reported no invalid swab results or did not report the data on invalid results.

Two studies were conducted in populations with an HIV prevalence of greater than 50%.^7^, ^23^ Shapiro and colleagues (tongue swab, COPAN FLOQSwabs, in-house PCR) had an HIV prevalence of 92% and showed a sensitivity of 66% (95% CI 53–77) and a specificity of 78% (95% CI 66–87) using a quantification cycle (Cq) threshold of 38.^7^ LaCourse and colleagues (buccal swab, Whatman OmniSwab, in-house PCR) had an HIV prevalence of 54%, a sensitivity of 68% (43–87), and a specificity of 83% (72–90; figure 3A).^23^

**Figure 4:**
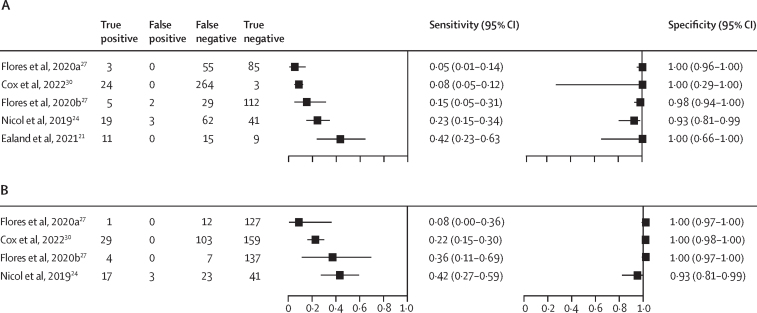
Forest plot of oral swabs in children using a composite (A) and microbiological (B) reference standard

Five studies (four reports, 815 participants) evaluated sensitivity of oral swabs as a specimen in children. Sensitivity in children ranged from 8% to 42% when using a microbiological reference standard, and 5% to 42% when using a composite reference standard (figure 4). Specificities ranged from 93% to 100%. Only one study in children reported invalid results, with four invalid swab results.^24^ Studies varied widely in terms of location of oral cavity swabbed, swab type, number of swabs collected, swab processing method, and molecular test used. All these factors impact sensitivity and specificity of oral swabs and molecular testing for the diagnosis of pulmonary tuberculosis. Given this, no meta-analysis of results was performed as studies differed too greatly.

## Discussion

In this systematic review of the diagnostic accuracy of oral swabs and molecular testing for pulmonary tuberculosis, we found that the sensitivity of oral swabs ranged from 36% to 91% for adults and between 5% and 42% for children. As expected, sensitivity was reduced in children who are more likely to have paucibacillary disease and an imperfect reference standard. Sensitivity of oral swabs as a clinical specimen varies in both adults and children when diverse methods are used. Specificity is less variable than sensitivity, with most studies reporting specificity greater than 90%.

Optimising sample processing methods, including swab type, anatomical location sampled, and NAAT platforms could lead to increased diagnostic accuracy with oral swabs. Luabeya and colleagues demonstrated that Cq values for MTB DNA were significantly lower (corresponding to stronger PCR signals) in samples collected from the tongue compared with the cheek or gingiva.^22^ Copan FLOQSwabs showed increased biomass collection relative to Whatman OmniSwabs and Puritan Purflock Ultra Swabs, which has been linked to higher sensitivity.^19^ Andama and colleagues in Uganda evaluated a protocol for tongue swabbing coupled with Xpert MTB/RIF Ultra using new sample processing methods. This was the first study in adults with presumptive tuberculosis to demonstrate high sensitivity of oral swabs using Xpert MTB/RIF Ultra. The authors found overall sensitivity of 72% (95% CI 59–83) and specificity of 100% (95% CI 97–100) using a microbiological reference standard,^21^ achieved using a double swabbing strategy and processing with Cepheid sample reagent and Xpert MTB/RIF Ultra. Using an alternative processing method, where a single swab was boiled, incubated, and mixed without using sample reagent, sensitivity was very similar at 77% (60–90) and specificity was 100% (16–100).^21^ With the increased biomass collected by double swabs, the processing method using Cepheid sample reagent showed similar sensitivity to the boil method (73% using the Cepheid sample reagent *vs* 77% using the boil method). The use of sample reagent and similar processing methods to sputum potentially allows laboratories already performing sputum testing with Xpert MTB/RIF Ultra to use oral swabs as a sample type with minimal change in processing methods.

Several other differences between studies included uses of different media types for swab transport, freezing of swab samples before testing versus immediate testing, and laboratory processing before PCR testing. These methods are continuously being evaluated but are not yet optimised. A consensus platform protocol for future research studies of oral swabs, which standardises procedures for swab collection, processing, storage, and other procedures, would improve consistency within studies, facilitate comparisons between studies, and allow more accurate accuracy estimates.

We were unable to perform meta-analyses as originally intended in the protocol because of considerable heterogeneity related to location of sampling, swab type, transport and storage methods, and type of NAAT used, as well as a limited number of studies for each combination of these factors (figure 1). A recent study meta-analysed several of the studies included in this review, and showed a summary sensitivity of 58·5% and specificity of 85·6% for oral swabs in the diagnosis of pulmonary tuberculosis.^25^ However, the sources of heterogeneity we identified were not investigated as part of that analysis.

Most studies included in this review had low risk of bias in all four QUADAS-2 domains. However, methods for evaluating accuracy of oral swabs were inconsistently described, which means some studies lack details of sample collection, transport, storage, and processing that are important to reproduce the results without substantial heterogeneity. This could lead to unrecognised sources of variability in sensitivity and specificity. In particular, there was frequent use of in-house PCR assays in many of the studies, which can vary considerably in specificity when compared with commercial PCR assays.^26^ In addition, many of these studies were conducted in populations with a high pre-test probability of disease and would be expected to have higher bacillary burdens, which is associated with increased sensitivity. Two studies showed that smear status and cavitary disease are associated with higher sensitivity.^17^, ^27^ Regarding applicability of our findings to the review question, in the index test domain, we considered applicability to be unclear as, currently, there is not a standardised protocol for oral swabs. The lack of a standardised protocol, with each study using a different approach, limited our ability to assess diagnostic accuracy by the methods evaluated in the studies.

The findings in this review were based on comprehensive searching, strict selection criteria, and standardised data extraction. To identify studies, we searched multiple databases up to July 5, 2022, without language restriction. However, we could have missed studies despite the comprehensive search. We corresponded with primary study authors to obtain additional data and information that was missing from the papers. In addition, this is a rapidly developing field, and a modified search update identified additional evidence published or submitted since the full search was completed on Aug 23, 2023. Although no additional studies using swabs have been published, additional studies^28^ have continued to explore saliva with findings of good sensitivity comparable with the highest sensitivities seen with oral swabs.

For detection of pulmonary tuberculosis, most studies evaluated oral swab specimens collected from participants with presumptive tuberculosis attending primary care facilities and local hospitals. Hence, for most studies, the participant characteristics and settings matched our review question. One study evaluated oral swab specimens as a screening test for pulmonary tuberculosis in an asymptomatic population at high risk for infection.^14^ Although this is a potential use of oral swabs for tuberculosis detection, it did not reflect the primary purpose of this review; further study in community-based settings and asymptomatic populations are needed to clarify these roles. Of note, since the majority of studies in adults required sputum to be collected for a microbiological reference standard, individuals unable to provide a sputum sample might have been underrepresented in this review. As sputum-scarce populations could particularly benefit from oral swab sampling, this is a limitation in the current literature on oral swabs which might misestimate the diagnostic yield of oral swabs in important target populations. As higher bacillary load does seem to predict increased sensitivity, it is crucial to optimise methods for sputum-scarce populations such as children and people living with HIV as they frequently present with lower pulmonary bacillary loads.

We found that sensitivity of oral swabs varies in both adults and children when diverse methods are used. Specificity is less variable than sensitivity with most studies reporting specificity greater than 90%. As oral swabs are not currently approved or recommended for use in the diagnosis of pulmonary tuberculosis, this review does not alter current clinical practice. We recommend that research going forward compares specific aspects of oral swabs, either through prospective cohort or clinical trials, so that optimal methods can be determined, such as direct comparisons of two swab types or of two processing methods such as seen in Andama and colleagues.^22^

Oral swabs with molecular testing can provide accurate results for the diagnosis of pulmonary tuberculosis. The highest sensitivities in this review approach the acceptable sensitivity range (as defined by WHO consolidated guidelines on tuberculosis^2^) for an initial diagnostic test relative to performance of sputum-based diagnostics, suggesting that with continued optimisation, swabs could be a good sample type for tuberculosis detection.^29^ Future studies should use a standardised protocol for comparing different aspects of oral swab collection and processing, keeping all aspects of the protocol the same except one. To advance the potential of oral swabs in a point-of-care diagnostic test, further study using existing point-of-care molecular WHO-recommended rapid diagnostics should be considered, as well as development of novel, low-complexity point-of-care platforms for use with oral swabs.

## Data sharing

The data included in this review are all published data or available by pre-print through authorised subscriptions. The protocol for this study is not available on any protocol storage site but is available on request from the corresponding author.

## Declaration of interests

This study received funding from FIND. ECC reports institution payments from FIND. MK and MR are employed by FIND. AES is supported in part by a National Institutes of Health (NIH) K23 AI40918 award. GAC is funded by the NIH and Bill and Melinda Gates foundation and reports receiving donations of research supplies (FLOQswabs) from Copan Italia. KS has received financial support from Cochrane Infectious Diseases, McGill University, Baylor College of Medicine, Maastricht University, TB Proof, and WHO Global Tuberculosis Programme; consultancy fees from FIND, the global alliance for diagnostics; consulting fees from Stellenbosch University, and travel support to attend WHO guideline development group meetings.
