# Supplementary material for: Oral swabs with a rapid molecular diagnostic test for pulmonary tuberculosis in adults and children: a systematic review
Source: Lancet Glob Health. 2023 Dec 12;12(1):e45–54. doi: 10.1016/S2214-109X(23)00469-2 (PMC10733129; doi:10.1016/S2214-109X(23)00469-2)
Supplement: Supplementary appendix [file mmc1.pdf]

THE LANCET
Global Health

## Supplementary appendix

This appendix formed part of the original submission and has been peer reviewed. We post it as supplied by the authors.

Supplement to: Church EC, Steingart KR, Cangelosi GA, Ruhwald M, Kohli M, Shapiro AE. Oral swabs with a rapid molecular diagnostic test for pulmonary tuberculosis in adults and children: a systematic review. *Lancet Glob Health* 2024; **12:** e45–54.

## Appendix

## Supplemental Methods: Search Strategy

Database: Ovid MEDLINE(R) and Epub Ahead of Print, In-Process, In-Data-Review & Other Non-Indexed Citations, Daily and Versions(R) <1946 to August 09, 2021>

Search Strategy:

--------------------------------------------------------------------------------

1 Mycobacterium tuberculosis/

2 Mycobacterium tuberculosis.tw.

3 (tuberculosis or TB).tw.

4 Tuberculosis, Pulmonary/

5 1 or 2 or 3 or 4

6 (oral swab* or Tongue scraping or mouth scraping).mp.

7 tongue.mp. or Tongue/

8 ((tongue or mouth or oral) adj2 swab*).tw.

9 buccal swab*.mp.

10 PurFlock.mp.

11 PrimeSwab.tw.

12 Copan FloqSwab*.tw.

13 "ORAL OMR 110".tw.

14 SLIM assay.mp.

15 6 or 7 or 8 or 9 or 10 or 11 or 12 or 13 or 14

16 5 and 15

17 limit 16 to humans

Database: Embase 1947-Present, updated daily

Search Strategy:

--------------------------------------------------------------------------------

1 Mycobacterium tuberculosis/

2 Mycobacterium tuberculosis.tw.

3 (tuberculosis or TB).tw.

4 lung tuberculosis/ or pulmonary tuberculosis.mp.

5 1 or 2 or 3 or 4

6 (oral swab* or Tongue scraping or mouth scraping).mp.

7 tongue.mp. or Tongue/

8 ((tongue or mouth or oral) adj2 swab*).tw.

9 buccal swab*.mp.

10 PurFlock.mp.

11 PrimeSwab.tw.

12 Copan FloqSwab*.tw.

13 "ORAL OMR 110".tw.

14 SLIM assay.mp.

15 6 or 7 or 8 or 9 or 10 or 11 or 12 or 13 or 14

16 5 and 15

17 limit 16 to human

18 limit 17 to yr="2000 -Current"

SCOPUS

( ( TITLE-ABS-KEY ( oral AND swab ) ) OR ( TITLE-ABS-KEY ( mouth AND swab ) ) OR ( TITLE-ABS-KEY ( tongue AND scraping ) ) OR ( TITLE-ABS-KEY ( mouth AND scraping ) ) OR ( TITLE-ABS-KEY ( tongue AND swab ) ) OR ( TITLE-ABS-KEY ( buccal AND swab ) ) OR ( TITLE-ABS-KEY ( buccal AND scraping ) ) OR ( TITLE-ABS-KEY ( purflock OR primeswab ) ) OR ( TITLE-ABS-KEY ( copan AND floqswab ) ) OR ( TITLE-ABS-KEY ( slim AND assay ) ) ) AND ( TITLE-ABS-KEY ( tuberculosis OR tb OR mycobacterium AND tuberculosis ) )

Global index medicus

(tw:(tuberculosis )) AND (tw:(PurFlock or PrimeSwab or Copan FloqSwab))

(tw:(mycobacterium )) AND (tw:(PurFlock or PrimeSwab or Copan FloqSwab))

(tw:(tuberculosis )) AND (tw:(oral swab or mouth swab or tongue scraping or mouth scraping))

Web of Science Core Collection Science ciation index-Expanded, Conference proceedings citation index

(((#4) OR #3) OR #2) OR #1

# 4   tuberculosis (Topic) and PurFlock or PrimeSwab or Copan FloqSwab* or "ORAL OMR 110" or "SLIM assay" (All Fields)

#3  mycobacterium (Topic) and PurFlock or PrimeSwab or Copan FloqSwab* or "ORAL OMR 110" or "SLIM assay" (All Fields)

#2 mycobacterium (Topic) and (oral or mouth or buccal or tongue) and (swab* or scraping) (All Fields)

#1 tuberculosis (Topic) and (oral or mouth or buccal or tongue) and (swab* or scraping) (All Fields)

medRxiv  and bioRxiv

tuberculosis  and PurFlock or PrimeSwab or Copan FloqSwab* or "ORAL OMR 110" or "SLIM assay"

## Supplemental Methods: QUADAS-2 Rules

Domain 1: patient selection

Risk of bias: could the selection of patients have introduced bias?

Signaling question 1: was a consecutive or random sample of patients enrolled? We will answer 'yes' if the study enrolled a consecutive or random sample of eligible patients; 'no' if the study selected patients by convenience; and 'unclear' if the study did not report the manner of patient selection or if there is insufficient information to make a decision

Signaling question 2: Was a case-control design avoided? Studies using a case-control design, especially when used to compare results in severely ill patients with those in relatively healthy individuals, may lead to overestimation of accuracy in diagnostic studies. We will answer 'yes' if the study avoided a case-control design, 'no' if the study used a case-control design, and unclear' if there is insufficient information to make a decision.

Signaling question 3: Did the study avoid inappropriate exclusions? We will answer "yes" if participants were primarily selected who were not previously on tuberculosis treatment. We will answer 'no' if the study included primarily or exclusively patients who had undergone previous tuberculosis treatment (retreatment patients) or included primarily patients with signs or symptoms of extrapulmonary tuberculosis.

Applicability: are there concerns that the included patients and setting do not match the review question?

We are interested in how oral swab analysis will perform in patients who are evaluated as they would be in intended usual practice. We will answer 'low concern' for most studies if participants were evaluated in primary care centers, non-healthcare settings, or local hospitals. We will answer 'unclear concern' if there is insufficient information to make a decision.

Domain 2: index test

Risk of bias: could the conduct or interpretation of the index test have introduced bias?

Signaling question 1: were the index test results interpreted without knowledge of the results of the reference standard? We will answer 'low concern' if the investigators were not aware of the reference standard results. We will answer 'high concern' if they were aware of the reference standard results. We will answer 'unclear concern' if there is insufficient information to make a decision.

Signaling question 2: if a threshold was used, was it prespecified? Threshold here applies to cycle threshold for PCR testing. We will answer 'low concern' if a threshold was prespecified or if no threshold was used. We will answer 'high concern' if the threshold was not prespecified. We will answer 'unclear concern' if there is insufficient information to make a decision.

Signaling question 3: Does the study provide sufficient details of all procedures carried out? We will answer “low concern” if methods contain sufficient detail to replicate sample collection, sample transport, and sample analysis. We will answer “high concern” if methods do not contain sufficient detail for replication of the above.

Applicability: are there concerns that the index test, its conduct, or its interpretation differ from the review question? Variations in test technology, execution, or interpretation may affect estimates of the diagnostic accuracy of a test. We will answer ‘unclear concern’ as there is no standardized method currently for the index test under evaluation.

Domain 3: reference standard

Risk of bias: could the reference standard, its conduct, or its interpretation have introduced bias?

Signaling question 1: is the reference standard likely to correctly classify the target condition?

For adults, we will answer ‘yes’ for all studies using a microbiological reference standard and unclear for studies using a composite refence standard. For children, we will answer yes for all studies using a microbiological or composite reference standard.

Signaling question 2: were the reference standard results interpreted without knowledge of the results of the index test?

We will answer ‘yes’ if the reference test provided an automated result, blinding was explicitly stated, or it was clear that the reference standard was performed at a separate laboratory and/or performed by different people. We will answer ‘no’ if the reference standard result was interpreted with knowledge of the swab-based NAAT test result. We will answer ‘unclear’ if there is insufficient information to make a decision.

Applicability: are there concerns that the target condition as defined by the reference standard does not match the question? We will answer ‘high concern’ if included studies did not identify the species of mycobacteria isolated in culture; or if only a composite reference standard was used in adult patients. ‘low concern’ if species identification was performed; or if a composite reference standard only was used in children, and ‘unclear concern’ if there is insufficient information to make a decision.

Domain 4: flow and timing

Risk of bias: could the patient flow have introduced bias?

Signaling question 1: was there an appropriate interval between the sample collection for the index test and reference standard? If there were a delay of several days between sample collection for the index test and reference standard, tuberculosis is a chronic disease and we considered misclassification of disease status to be unlikely, as long as treatment was not initiated in the interim. We will answer ‘yes’ if sample collection for the index test and reference standard were performed at the same time or if the

time interval was less than or equal to seven days, ‘no’ if the time interval was greater than seven days, and ‘unclear’ if there is insufficient information to make a decision.

Signaling question 2: did all patients receive the same reference standard? We will answer this question ‘yes’ for all studies as an acceptable reference was specified as a criterion for inclusion in the review.

Signaling question 3: were all patients included in the analysis? We will determine the answer to this question by comparing the number of patients enrolled with the number of patients included in the 2 x 2 tables. We will answer ‘yes’ if the numbers matched and ‘no’ if there were patients enrolled in the study that were not included in the analysis and did not have a reported test reason for not being included (such as contaminate/lost specimen). We will answer ‘unclear’ if there is insufficient information to make a decision.

Judgements for ‘Risk of bias’ assessments for a given domain
If we answer all signaling questions for a domain ‘yes’, then we will judge risk of bias as ‘low’.
If we answer all or most signaling questions for a domain ‘no’, then we will judge risk of bias as ‘high’.
If we answer only one signaling question for a domain ‘no’, we will discuss further the risk of bias judgement.
If we answer all or most signaling questions for a domain ‘unclear’, then we will judge risk of bias as ‘unclear’.
If we answer only one signaling question for a domain ‘unclear’, we will discuss further the risk of bias judgement for the domain.

## Supplemental Results: Methodological Qualities of reviewed Studies

In the Patient Selection domain, we considered 13 studies (65%) to have low risk of bias because the study enrolled a consecutive or random sample of eligible participants, avoided a case-control design, and avoided inappropriate exclusions[7,19-24,27,30-32]. We considered seven studies (35%) to have a high risk of bias because the study did not avoid a case-control design[14-19]. With respect to applicability, we considered 19 studies (95%) to have low concern because participants in these studies were evaluated in primary care facilities, local hospitals, or both settings[7,14-20,22-24,27,30-32]. We considered 1 study (5%) to have high concern because participants were evaluated exclusively as inpatients in tertiary care centers[21].

In the Index Test domain, we considered 19 studies to have low risk of bias because the researchers were blinded to the results of the reference test, the threshold for a positive test was pre-specified (where applicable), and the study provided sufficient details of all procedures carried out[7,14-19,21-24,27,30-32]. We considered 1 study to have high risk of bias due to lack of a pre-specified threshold for test positivity and insufficient details of all procedures carried out[20]. With respect to applicability, we considered all but one study to have unclear concern because there is currently no standard methodology for oral swabs for the diagnosis of pulmonary tuberculosis. Lima et all was considered high concern, as the index test was used for screening rather than diagnostic purposes[14].

In the Reference Standard domain, we considered all studies to have low risk of bias because studies in adults included a sputum microbiological reference standard and studies in children included a microbiological or clinical reference standard and the results of the reference standard were interpreted without knowledge of the results of the index test. With respect to applicability, we considered all studies to have low concern as studies identified mycobacterial species in culture and all studies collected sputum samples for microbiological (mWRD or culture) testing.

In the Flow and Timing domain, we considered 18 studies (90%) to have low risk of bias because all participants were included in the analysis, all participants had the same reference standard, and there was an appropriate interval between index and reference standard collection (< 7 days) [7,14-24,30-32]. We considered 2 studies (10%) to have an unclear risk of bias as the timing of index and reference standard samples was not specified[27].

Supplemental Figure 1. Sample clinical pathway for oral swabs in the diagnosis of pulmonary tuberculosis

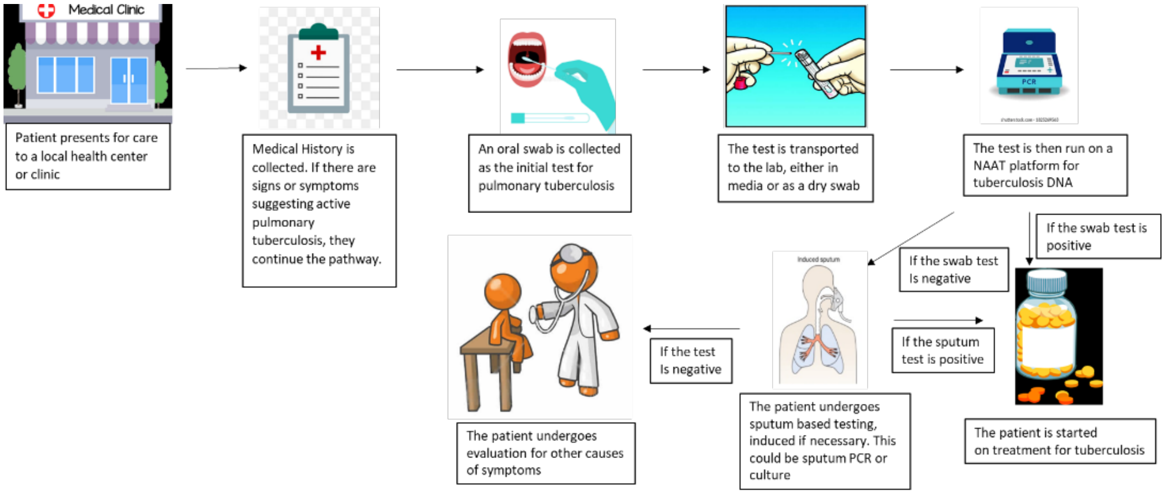


• True-positive: patients would benefit from rapid diagnosis and initiation of appropriate treatment.
• True-negative: patients would have to undergo additional testing
for alternative diagnoses. TN patients would avoid unnecessary treatment
• False-positive: patients may experience anxiety and morbidity caused by additional testing, unnecessary treatment,
or adverse events; stigma associated with a TB or MDR-TB diagnosis; and the chance that a false-positive result may halt further diagnostic evaluation.
• False-negative: patients would experience a delay in treatment while additional diagnostic procedures are performed.

## Supplemental Figure 2

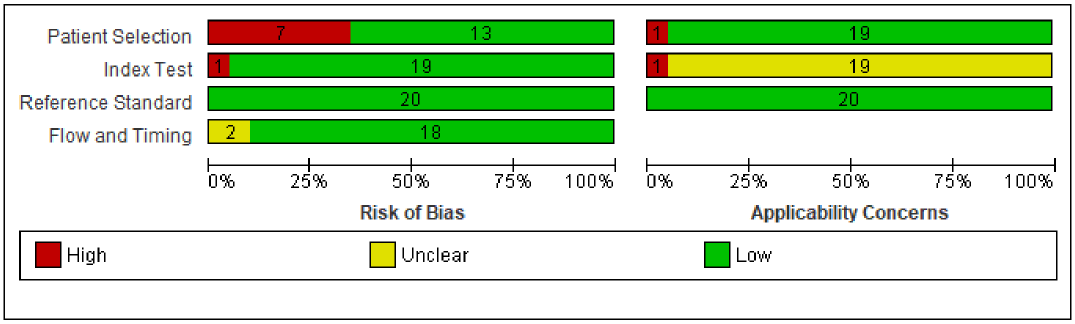


Risk of bias and applicability concerns graph: review authors' judgements about each domain presented as percentages across included studies

## Supplemental Figure 3

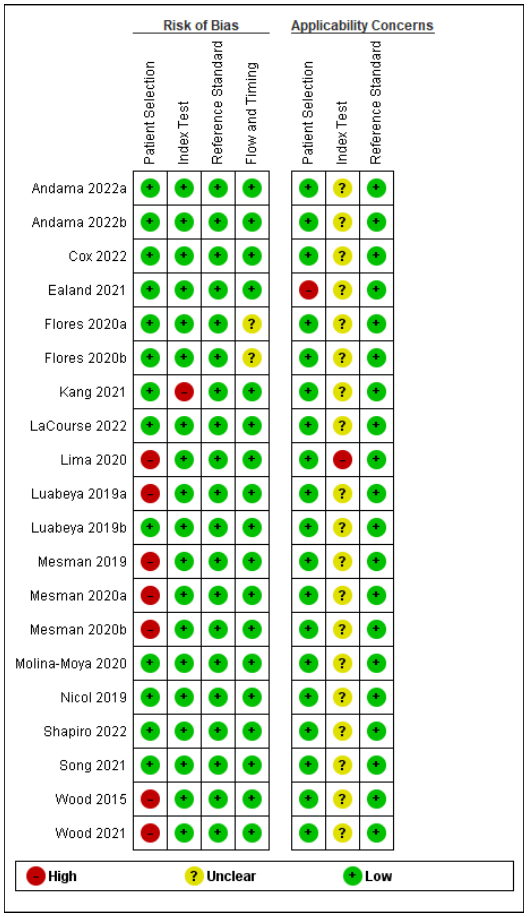


Risk of bias and applicability concerns summary: review authors' judgements about each domain for each included

Supplemental Figure 4. Forest plot of oral swabs in adults and children using a microbiological reference standard

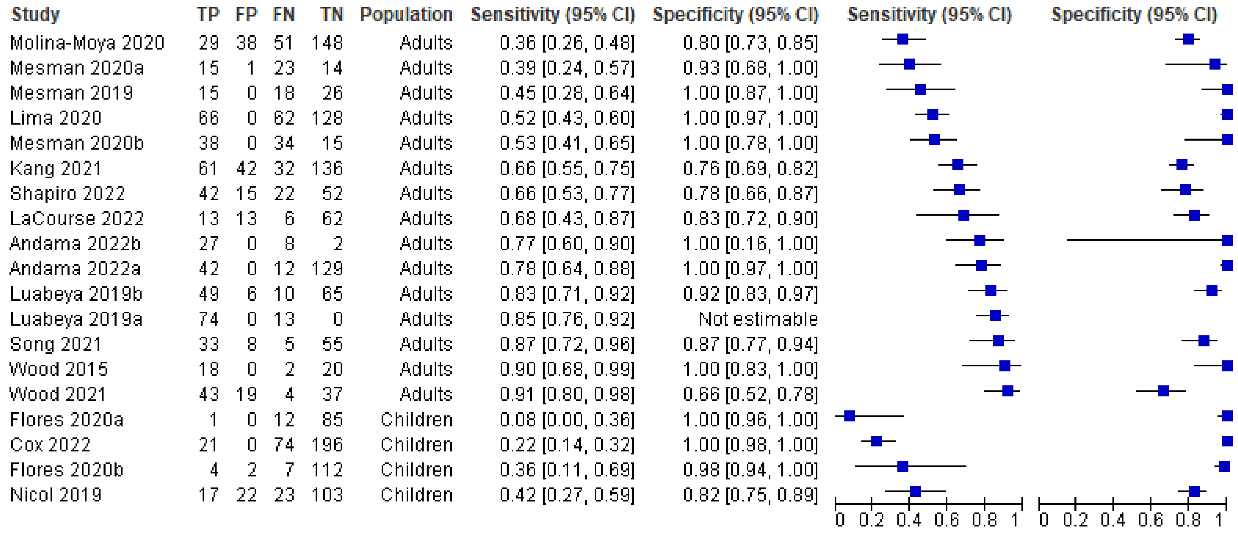

| Study | TP | FP | FN | TN | Population | Sensitivity (95% CI) | Specificity (95% CI) |
|---|---|---|---|---|---|---|---|
| Molina-Moya 2020 | 29 | 38 | 51 | 148 | Adults | 0.36 [0.26, 0.48] | 0.80 [0.73, 0.85] |
| Mesman 2020a | 15 | 1 | 23 | 14 | Adults | 0.39 [0.24, 0.57] | 0.93 [0.68, 1.00] |
| Mesman 2019 | 15 | 0 | 18 | 26 | Adults | 0.45 [0.28, 0.64] | 1.00 [0.87, 1.00] |
| Lima 2020 | 66 | 0 | 62 | 128 | Adults | 0.52 [0.43, 0.60] | 1.00 [0.97, 1.00] |
| Mesman 2020b | 38 | 0 | 34 | 15 | Adults | 0.53 [0.41, 0.65] | 1.00 [0.78, 1.00] |
| Kang 2021 | 61 | 42 | 32 | 136 | Adults | 0.66 [0.55, 0.75] | 0.76 [0.69, 0.82] |
| Shapiro 2022 | 42 | 15 | 22 | 52 | Adults | 0.66 [0.53, 0.77] | 0.78 [0.66, 0.87] |
| LaCourse 2022 | 13 | 13 | 6 | 62 | Adults | 0.68 [0.43, 0.87] | 0.83 [0.72, 0.90] |
| Andama 2022b | 27 | 0 | 8 | 2 | Adults | 0.77 [0.60, 0.90] | 1.00 [0.16, 1.00] |
| Andama 2022a | 42 | 0 | 12 | 129 | Adults | 0.78 [0.64, 0.88] | 1.00 [0.97, 1.00] |
| Luabeya 2019b | 49 | 6 | 10 | 65 | Adults | 0.83 [0.71, 0.92] | 0.92 [0.83, 0.97] |
| Luabeya 2019a | 74 | 0 | 13 | 0 | Adults | 0.85 [0.76, 0.92] | Not estimable |
| Song 2021 | 33 | 8 | 5 | 55 | Adults | 0.87 [0.72, 0.96] | 0.87 [0.77, 0.94] |
| Wood 2015 | 18 | 0 | 2 | 20 | Adults | 0.90 [0.68, 0.99] | 1.00 [0.83, 1.00] |
| Wood 2021 | 43 | 19 | 4 | 37 | Adults | 0.91 [0.80, 0.98] | 0.66 [0.52, 0.78] |
| Flores 2020a | 1 | 0 | 12 | 85 | Children | 0.08 [0.00, 0.36] | 1.00 [0.96, 1.00] |
| Cox 2022 | 21 | 0 | 74 | 196 | Children | 0.22 [0.14, 0.32] | 1.00 [0.98, 1.00] |
| Flores 2020b | 4 | 2 | 7 | 112 | Children | 0.36 [0.11, 0.69] | 0.98 [0.94, 1.00] |
| Nicol 2019 | 17 | 22 | 23 | 103 | Children | 0.42 [0.27, 0.59] | 0.82 [0.75, 0.89] |

Supplemental Figure 5. Forest plot of oral swabs in adults using a microbiological reference standard by swab type

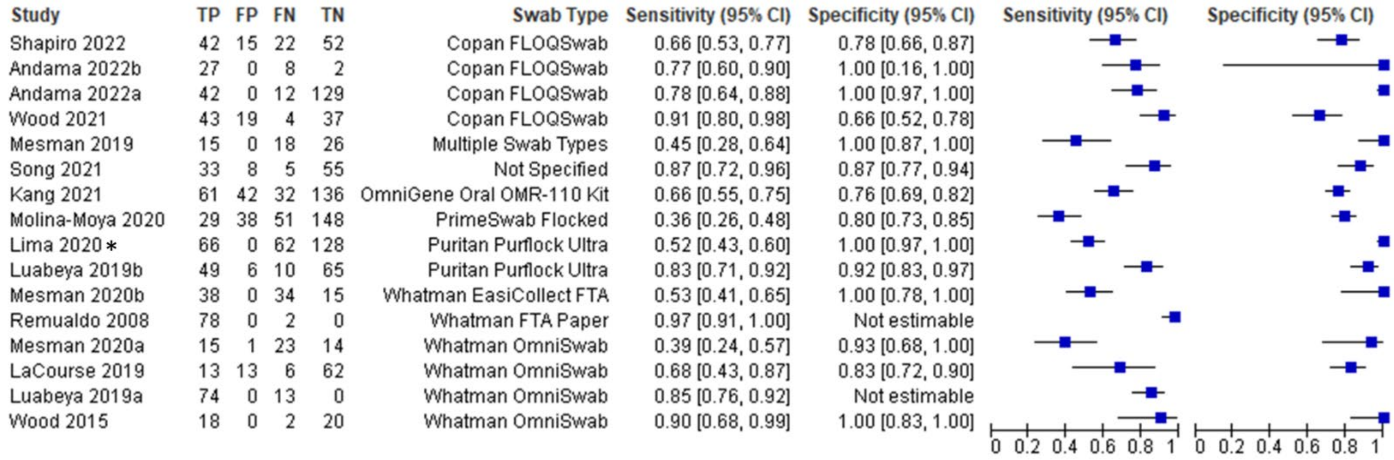

| Study | TP | FP | FN | TN | Swab Type | Sensitivity (95% CI) | Specificity (95% CI) |
|---|---|---|---|---|---|---|---|
| Shapiro 2022 | 42 | 15 | 22 | 52 | Copan FLOQSwab | 0.66 [0.53, 0.77] | 0.78 [0.66, 0.87] |
| Andama 2022b | 27 | 0 | 8 | 2 | Copan FLOQSwab | 0.77 [0.60, 0.90] | 1.00 [0.16, 1.00] |
| Andama 2022a | 42 | 0 | 12 | 129 | Copan FLOQSwab | 0.78 [0.64, 0.88] | 1.00 [0.97, 1.00] |
| Wood 2021 | 43 | 19 | 4 | 37 | Copan FLOQSwab | 0.91 [0.80, 0.98] | 0.66 [0.52, 0.78] |
| Mesman 2019 | 15 | 0 | 18 | 26 | Multiple Swab Types | 0.45 [0.28, 0.64] | 1.00 [0.87, 1.00] |
| Song 2021 | 33 | 8 | 5 | 55 | Not Specified | 0.87 [0.72, 0.96] | 0.87 [0.77, 0.94] |
| Kang 2021 | 61 | 42 | 32 | 136 | OmniGene Oral OMR-110 Kit | 0.66 [0.55, 0.75] | 0.76 [0.69, 0.82] |
| Molina-Moya 2020 | 29 | 38 | 51 | 148 | PrimeSwab Flocked | 0.36 [0.26, 0.48] | 0.80 [0.73, 0.85] |
| Lima 2020* | 66 | 0 | 62 | 128 | Puritan Purflock Ultra | 0.52 [0.43, 0.60] | 1.00 [0.97, 1.00] |
| Luabeya 2019b | 49 | 6 | 10 | 65 | Puritan Purflock Ultra | 0.83 [0.71, 0.92] | 0.92 [0.83, 0.97] |
| Mesman 2020b | 38 | 0 | 34 | 15 | Whatman EasiCollect FTA | 0.53 [0.41, 0.65] | 1.00 [0.78, 1.00] |
| Remualdo 2008 | 78 | 0 | 2 | 0 | Whatman FTA Paper | 0.97 [0.91, 1.00] | Not estimable |
| Mesman 2020a | 15 | 1 | 23 | 14 | Whatman OmniSwab | 0.39 [0.24, 0.57] | 0.93 [0.68, 1.00] |
| LaCourse 2019 | 13 | 13 | 6 | 62 | Whatman OmniSwab | 0.68 [0.43, 0.87] | 0.83 [0.72, 0.90] |
| Luabeya 2019a | 74 | 0 | 13 | 0 | Whatman OmniSwab | 0.85 [0.76, 0.92] | Not estimable |
| Wood 2015 | 18 | 0 | 2 | 20 | Whatman OmniSwab | 0.90 [0.68, 0.99] | 1.00 [0.83, 1.00] |

*This study used oral swabs as a screening tool in asymptomatic participants. All other studies were performed using oral swabs as a diagnostic test sample.

Supplemental Figure 6. Forest plot of orals swabs in adults using a microbiological reference standard by type of NAAT used

| Study | TP | FP | FN | TN | Type of NAAT | Sensitivity (95% CI) | Specificity (95% CI) |
|---|---|---|---|---|---|---|---|
| Molina-Moya 2020 | 29 | 38 | 51 | 148 | In-house PCR | 0.36 [0.26, 0.48] | 0.80 [0.73, 0.85] |
| Mesman 2020a | 15 | 1 | 23 | 14 | In-house PCR | 0.39 [0.24, 0.57] | 0.93 [0.68, 1.00] |
| Mesman 2020b | 38 | 0 | 34 | 15 | In-house PCR | 0.53 [0.41, 0.65] | 1.00 [0.78, 1.00] |
| Shapiro 2022 | 42 | 15 | 22 | 52 | In-house PCR | 0.66 [0.53, 0.77] | 0.78 [0.66, 0.87] |
| LaCourse 2022 | 13 | 13 | 6 | 62 | In-house PCR | 0.68 [0.43, 0.87] | 0.83 [0.72, 0.90] |
| Luabeya 2019b | 49 | 6 | 10 | 65 | In-house PCR | 0.83 [0.71, 0.92] | 0.92 [0.83, 0.97] |
| Luabeya 2019a | 74 | 0 | 13 | 0 | In-house PCR | 0.85 [0.76, 0.92] | Not estimable |
| Wood 2015 | 18 | 0 | 2 | 20 | In-house PCR | 0.90 [0.68, 0.99] | 1.00 [0.83, 1.00] |
| Wood 2021 | 43 | 19 | 4 | 37 | In-house PCR | 0.91 [0.80, 0.98] | 0.66 [0.52, 0.78] |
| Song 2021 | 33 | 8 | 5 | 55 | TB LAMP Assay | 0.87 [0.72, 0.96] | 0.87 [0.77, 0.94] |
| Mesman 2019 | 15 | 0 | 18 | 26 | Xpert MTB/RIF Ultra | 0.45 [0.28, 0.64] | 1.00 [0.87, 1.00] |
| Lima 2020 | 66 | 0 | 62 | 128 | Xpert MTB/RIF Ultra | 0.52 [0.43, 0.60] | 1.00 [0.97, 1.00] |
| Andama 2022b | 27 | 0 | 8 | 2 | Xpert MTB/RIF Ultra | 0.77 [0.60, 0.90] | 1.00 [0.16, 1.00] |
| Andama 2022a | 42 | 0 | 12 | 129 | Xpert MTB/RIF Ultra | 0.78 [0.64, 0.88] | 1.00 [0.97, 1.00] |

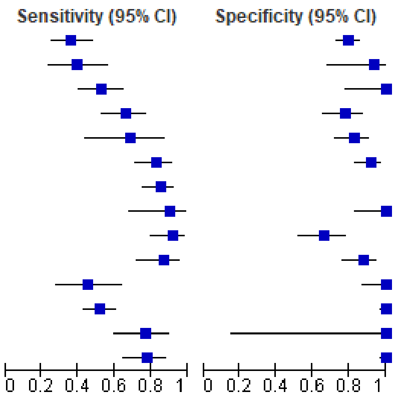

# PRISMA-DTA for Abstracts Checklist

| Section/topic | # | PRISMA-DTA for Abstracts Checklist item | Reported on page # |
|---|---|---|---|
| **TITLE and PURPOSE** | | | |
| Title | 1 | Identify the report as a systematic review (+/- meta-analysis) of diagnostic test accuracy (DTA) studies. | 1 |
| Objectives | 2 | Indicate the research question, including components such as participants, index test, and target conditions. | 1 |
| **METHODS** | | | |
| Eligibility criteria | 3 | Include study characteristics used as criteria for eligibility. | 1 |
| Information sources | 4 | List the key databases searched and the search dates. | 1 |
| Risk of bias & applicability | 5 | Indicate the methods of assessing risk of bias and applicability. | 1 |
| Synthesis of results | A1 | Indicate the methods for the data synthesis. | 1 |
| **RESULTS** | | | |
| Included studies | 6 | Indicate the number and type of included studies and the participants and relevant characteristics of the studies (including the reference standard). | 1 |
| Synthesis of results | 7 | Include the results for the analysis of diagnostic accuracy, preferably indicating the number of studies and participants. Describe test accuracy including variability; if meta-analysis was done, include summary results and confidence intervals. | 1 |
| **DISCUSSION** | | | |
| Strengths and limitations | 9 | Provide a brief summary of the strengths and limitations of the evidence | 1 |
| Interpretation | 10 | Provide a general interpretation of the results and the important implications. | 1 |
| **OTHER** | | | |
| Funding | 11 | Indicate the primary source of funding for the review. | 1 |
| Registration | 12 | Provide the registration number and the registry name | N/A |

*Adapted From:* McInnes MDF, Moher D, Thombs BD, McGrath TA, Bossuyt PM, The PRISMA-DTA Group (2018). Preferred Reporting Items for a Systematic Review and Meta-analysis of Diagnostic Test Accuracy Studies: The PRISMA-DTA Statement. JAMA. 2018 Jan 23;319(4):388-396. doi: 10.1001/jama.2017.19163.

For more information, visit: **www.prisma-statement.org**.

# PRISMA-DTA Checklist

| Section/topic | # | PRISMA-DTA Checklist Item | Reported on page # |
|---|---|---|---|
| **TITLE / ABSTRACT** | | | |
| Title | 1 | Identify the report as a systematic review (+/- meta-analysis) of diagnostic test accuracy (DTA) studies. | 1 |
| Abstract | 2 | Abstract: See PRISMA-DTA for abstracts. | 1 |
| **INTRODUCTION** | | | |
| Rationale | 3 | Describe the rationale for the review in the context of what is already known. | 3 |
| Clinical role of index test | D1 | State the scientific and clinical background, including the intended use and clinical role of the index test, and if applicable, the rationale for minimally acceptable test accuracy (or minimum difference in accuracy for comparative design). | 3 |
| Objectives | 4 | Provide an explicit statement of question(s) being addressed in terms of participants, index test(s), and target condition(s). | 3 |
| **METHODS** | | | |
| Protocol and registration | 5 | Indicate if a review protocol exists, if and where it can be accessed (e.g., Web address), and, if available, provide registration information including registration number. | N/A |
| Eligibility criteria | 6 | Specify study characteristics (participants, setting, index test(s), reference standard(s), target condition(s), and study design) and report characteristics (e.g., years considered, language, publication status) used as criteria for eligibility, giving rationale. | 3 |
| Information sources | 7 | Describe all information sources (e.g., databases with dates of coverage, contact with study authors to identify additional studies) in the search and date last searched. | 3 |
| Search | 8 | Present full search strategies for all electronic databases and other sources searched, including any limits used, such that they could be repeated. | 3 |
| Study selection | 9 | State the process for selecting studies (i.e., screening, eligibility, included in systematic review, and, if applicable, included in the meta-analysis). | 4 |
| Data collection process | 10 | Describe method of data extraction from reports (e.g., piloted forms, independently, in duplicate) and any processes for obtaining and confirming data from investigators. | 4 |
| Definitions for data extraction | 11 | Provide definitions used in data extraction and classifications of target condition(s), index test(s), reference standard(s) and other characteristics (e.g. study design, clinical setting). | 4 |
| Risk of bias and applicability | 12 | Describe methods used for assessing risk of bias in individual studies and concerns regarding the applicability to the review question. | 4 |
| Diagnostic accuracy measures | 13 | State the principal diagnostic accuracy measure(s) reported (e.g. sensitivity, specificity) and state the unit of assessment (e.g. per-patient, per-lesion). | 4 |
| Synthesis of results | 14 | Describe methods of handling data, combining results of studies and describing variability between studies. This could include, but is not limited to: a) handling of multiple definitions of target condition. b) handling of multiple thresholds of test positivity, c) handling multiple index test readers, d) handling of indeterminate test results, e) grouping and comparing tests, f) handling of different reference standards | 4 |

# PRISMA-DTA Checklist

| Section/topic | # | PRISMA-DTA Checklist Item | Reported on page # |
|---|---|---|---|
| Meta-analysis | D2 | Report the statistical methods used for meta-analyses, if performed. | N/A |
| Additional analyses | 16 | Describe methods of additional analyses (e.g., sensitivity or subgroup analyses, meta-regression), if done, indicating which were pre-specified. | N/A |
| **RESULTS** | | | |
| Study selection | 17 | Provide numbers of studies screened, assessed for eligibility, included in the review (and included in meta-analysis, if applicable) with reasons for exclusions at each stage, ideally with a flow diagram. | 4 |
| Study characteristics | 18 | For each included study provide citations and present key characteristics including: a) participant characteristics (presentation, prior testing), b) clinical setting, c) study design, d) target condition definition, e) index test, f) reference standard, g) sample size, h) funding sources | 4, Table 1 |
| Risk of bias and applicability | 19 | Present evaluation of risk of bias and concerns regarding applicability for each study. | 5 |
| Results of individual studies | 20 | For each analysis in each study (e.g. unique combination of index test, reference standard, and positivity threshold) report 2x2 data (TP, FP, FN, TN) with estimates of diagnostic accuracy and confidence intervals, ideally with a forest or receiver operator characteristic (ROC) plot. | 4, figures 3 and 4 |
| Synthesis of results | 21 | Describe test accuracy, including variability; if meta-analysis was done, include results and confidence intervals. | 6 |
| Additional analysis | 23 | Give results of additional analyses, if done (e.g., sensitivity or subgroup analyses, meta-regression; analysis of index test: failure rates, proportion of inconclusive results, adverse events). | 6 |
| **DISCUSSION** | | | |
| Summary of evidence | 24 | Summarize the main findings including the strength of evidence. | 6 |
| Limitations | 25 | Discuss limitations from included studies (e.g. risk of bias and concerns regarding applicability) and from the review process (e.g. incomplete retrieval of identified research). | 6, 7 |
| Conclusions | 26 | Provide a general interpretation of the results in the context of other evidence. Discuss implications for future research and clinical practice (e.g. the intended use and clinical role of the index test). | 7 |
| **FUNDING** | | | |
| Funding | 27 | For the systematic review, describe the sources of funding and other support and the role of the funders. | 8 |

*Adapted From:* McInnes MDF, Moher D, Thombs BD, McGrath TA, Bossuyt PM, The PRISMA-DTA Group (2018). Preferred Reporting Items for a Systematic Review and Meta-analysis of Diagnostic Test Accuracy Studies: The PRISMA-DTA Statement. JAMA. 2018 Jan 23;319(4):388-396. doi: 10.1001/jama.2017.19163.

For more information, visit: **www.prisma-statement.org**.
